# Quack Medicines

**Published:** 1920-08-07

**Authors:** 


**THE PUBLIC WELL-BEING**—(*continued*).

### Quack Medicines.

### A BILL TO SAFEGUARD THE PUBLIC.

It is quite likely that the Proprietary Medicines Bill, introduced in the House of Lords by Lord Astor (and referred to in our leading columns), will excite a good deal of opposition from interested parties, but we hope that for the security of the public its main clauses will be retained without substantial alteration. It is common knowledge among members of the medical profession, as well as among all people concerned in questions of public health, that the indiscriminate sale of quack medicines is a source of real danger to the community. Unintelligent and credulous persons are readily attracted by advertisements which plausibly promise a quick cure for their ailments. The result is that without attempting to obtain qualified advice they spend money which they can ill afford to lose in experimenting with all kinds of useless nostrums which may in many cases be positively harmful and aggravate their complaints. Even more serious still is the fact that their ignorant delusions frequently prevent them from seeking for diagnosis and treatment in the right quarter until the disease has reached such an advanced stage that the chances of remedy or recovery are greatly diminished or completely destroyed.

This growing evil was rightly apprehended by the House of Commons Select Committee which reported on the subject last year. That Committee, after hearing a vast amount of evidence, found that there was a large and increasing sale in this country of patent and proprietary remedies and appliances and medicated wines, and that these remedies were of a widely different character: (*a*) Genuine scientific preparations, (*b*) unobjectionable remedies for simple ailments, and (*c*) many secret remedies, making grossly exaggerated claims of efficacy, causing injury by leading sick persons to delay in securing medical treatment, containing in disguise large proportions of alcohol sold for improper medication, professing to cure diseases incurable by medication, or essentially or deliberately fraudulent. It was at the last class that the Committee directed a brisk artillery fire, and urgently recommended a drastic amendment of the law.

The new Bill is in its main particulars the logical outcome of the Committee's report. It is proposed that all manufacturers and vendors of proprietary medicines or surgical appliances shall be registered, as well as the medicine and appliance, and that the medicine shall be compounded of the ingredients and in the proportions specified and the appliance correspond with the specimen furnished. Registration is to be signified on the article for sale by a number. The second clause lays down that it shall be unlawful to sell any medicine or appliance purporting to prevent, cure, or relieve a number of important diseases set out in a schedule, which can be extended by the Minister of Health at discretion. Prohibition is also made against the adver-

**THE PUBLIC WELL-BEING—(*continued*)**

tisement of any article that might be used for abortion or miscarriage. The third clause prohibits invitations by correspondence to take up remedies—in our view an extremely valuable provision; the use of misleading or fictitious testimonials; the issue of a statement that a medicine or appliance is recommended by a duly qualified medical practitioner without including in the particulars, names, qualifications, and the address of the person referred to; and the publication of a false statement that a medicine or appliance was discovered, invented, or compounded by a medical practitioner.

There are several other salutary provisions, and severe penalties are imposed for any infringement of the law. We hope the Ministry of Health will have the courage to carry through the Bill in the face of any trade opposition, direct or indirect, that may be forthcoming, because we believe the measure will have the effect of checking and finally stamping out a grave public evil that has been allowed too long to flourish openly. The natural antidote to the patent medicine is the panel doctor; and no member of the public, however poverty-stricken, can, in these days, complain that in the absence of the many decorative but questionable fancy medicines of the chemist's shop he will be deprived of a prompt and inexpensive means of medical assistance.

